# Supplementary material for: Interface-driven energy-independent charge extraction in GaN photocatalysts
Source: Nat Commun. 2026 Feb 18;17:1853. doi: 10.1038/s41467-026-69683-8 (PMC12920748; doi:10.1038/s41467-026-69683-8)
Supplement: Supplementary file 1 — Supplementary Information [file 41467_2026_69683_MOESM1_ESM.pdf]

<u>**Supplementary Information**</u>

Interface-driven energy-independent charge extraction in GaN photocatalysts

Yuying Gao[1,2,#,*], Yuxin Xie[2,3,#], Christian Höhn[1], Markus Wollgarten[1], Holger Kropf[1], Fengtao Fan[2], Can Li[2], Roel van de Krol[1,4], Dennis Friedrich[1,*]

[1]Institute for Solar Fuels, Helmholtz-Zentrum Berlin für Materialien und Energie GmbH, Berlin 14109, Germany.

[2]State Key Laboratory of Catalysis, Dalian National Laboratory for Clean Energy, Dalian Institute of Chemical Physics, Chinese Academy of Sciences, Dalian 116023, China.

[3]School of Chemistry and Materials Science, University of Science and Technology of China, Hefei 230026, China.

[4]Institut für Chemie, Technische Universität Berlin, Berlin 10623, Germany

[#]These authors contributed equally: Yuying Gao, Yuxin Xie.

[*]Corresponding author:

Yuying Gao: yuying.gao@helmholtz-berlin.de

Dennis Friedrich: friedrich@helmholtz-berlin.de

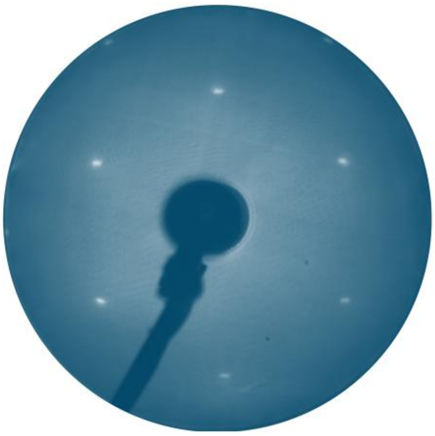

Figure S1. LEED patterns for n-GaN (0001) after surface clean treatment. The image was taken at an energy of 226 eV. Upon three cycles of Ar^+ sputtering and annealing (850℃ for 30 min) under UHV conditions, the GaN (0001) surface displays a well-defined (1×1) LEED diffraction pattern with hexagonal symmetry, indicative of a highly ordered surface structure. This observation suggests the formation of a Ga adlayer atop the N-terminated GaN surface.

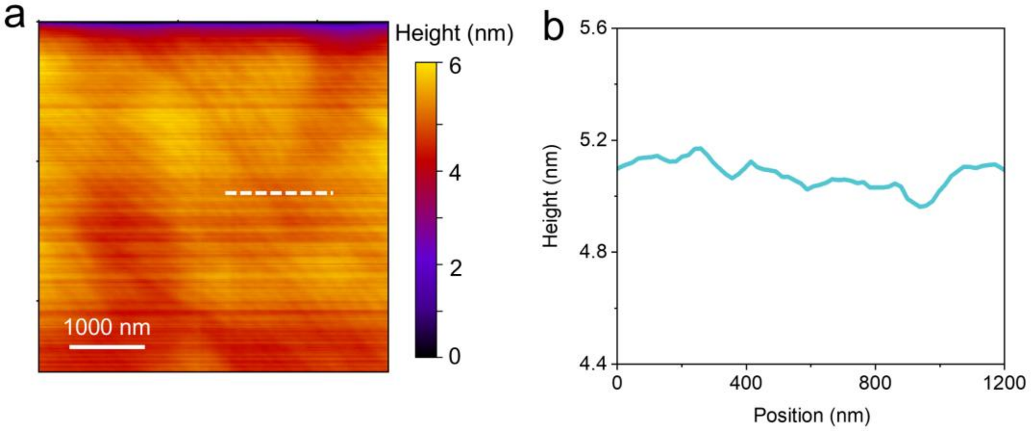

Figure S2. (a) AFM topography of the GaN wafer fabricated by hydride vapor phase epitaxy (HVPE). (b) Height profile of GaN surface along the white line indicated in a. The surface

height variation originates from the film edges or step sites. The surface roughness is less than 0.2 nm.

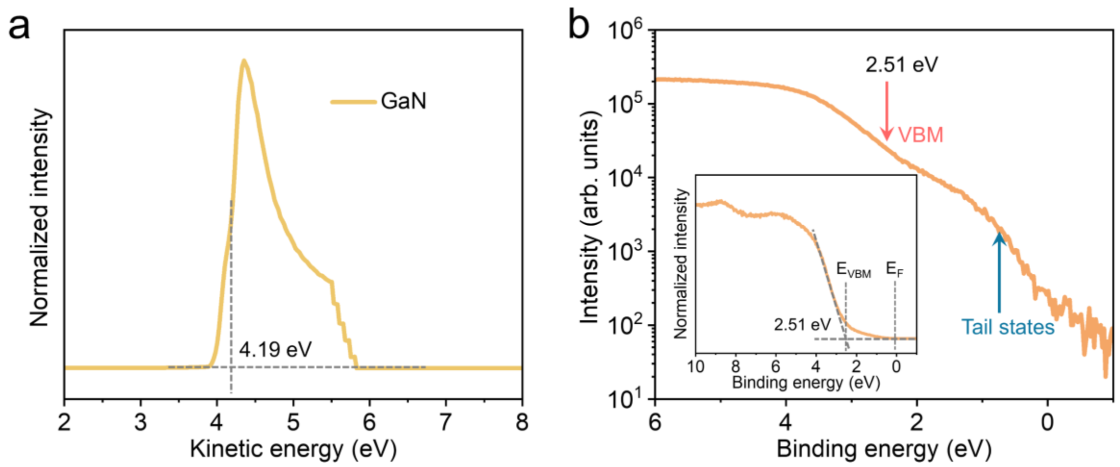

Figure S3. (a) Work function edges in UPS spectra of n-GaN wafer measured at a sample bias of -5 V. (b) Valence band spectroscopy of the GaN sample displayed in both logarithmic and linear scales (inset). GaN valence band maximum (VBM) feature is evident in the linear scale. The VBM is located 2.51 eV below the Fermi level, positioning the conduction band minimum (CBM) 0.89 eV above the Fermi level. The defect-induced surface states create a continuous band reaching the Fermi level.

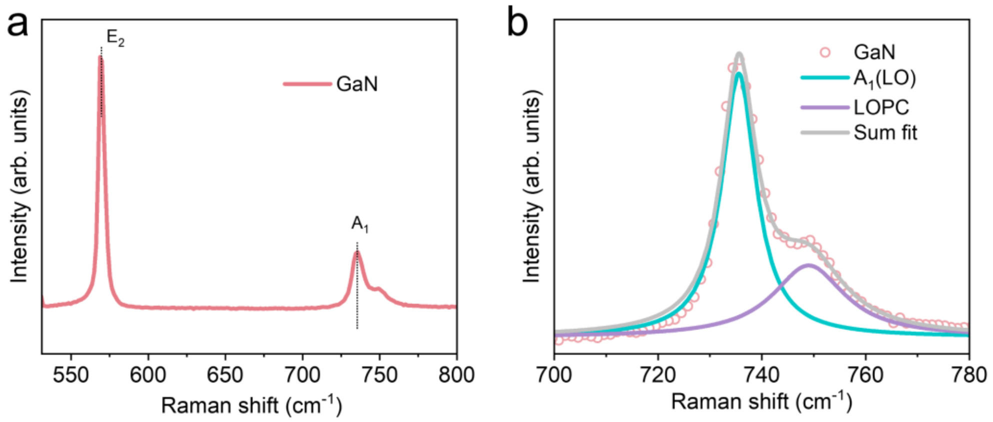

Figure S4. (a) Raman spectra of GaN sample exhibit a strong symmetric E_2 mode (568 cm^{-1}) and an asymmetric feature around 744 cm^{-1}. This asymmetric signal is attributed to the A_1 (LO) mode (735 cm^{-1}) coupled with high frequency plasmon mode, which is associated with weakly *n*-type GaN surface at relatively low Mg doping condition. (b) Raman signal (open circles) in the high-frequency LO-plasmon coupled (LOPC) region for the GaN sample. The solid lines represent Lorentzian fits, and the grey line denotes the sum of the two fitted components.

As the strong coupling between the longitudinal optical (LO) phonons and the collective oscillations of free carriers (plasmons) leads to the formation of the LO phonon-plasmon coupled (LOPC) mode, the A_1 (LO) phonon mode can serve as an effective probe to measure the free carrier concentration in GaN[1,2]. Notably, the relationship between plasmon frequency and the free carrier concentration can be expressed by:

$$\omega_p^2 = \frac{4\pi e^2 n_c}{m_c^* \epsilon_\infty}$$

where ω_p is the plasmon frequency, n_c is the free carrier concentration, m_c^*=0.22m_0 is the conduction band effective mass of free electron, ϵ_∞ is the high frequency dielectric constant. The plasmon frequency (ω_p) was calculated based on the equation[1]:

$$\omega_{LOPC}^2 = 0.5 \times \left\{\omega_{A_1}^2 + \omega_p^2 + \left[\left(\omega_{A_1}^2 + \omega_p^2\right)^2 - 4\omega_{A_1(TO)}^2\omega_p^2\right]^{0.5}\right\}$$

where ω_{LOPC}=749 cm^{-1} is the coupled plasmon frequency obtained from fitting results, ω_{A_1}= 735 cm^{-1} and $\omega_{A_1(TO)}$=532 cm^{-1} are the frequencies of the A1 (LO) and A1 (TO) modes. Hence, the free carrier concentration for the GaN is calculated to be 6.2 ×10^{16} cm^{3}.

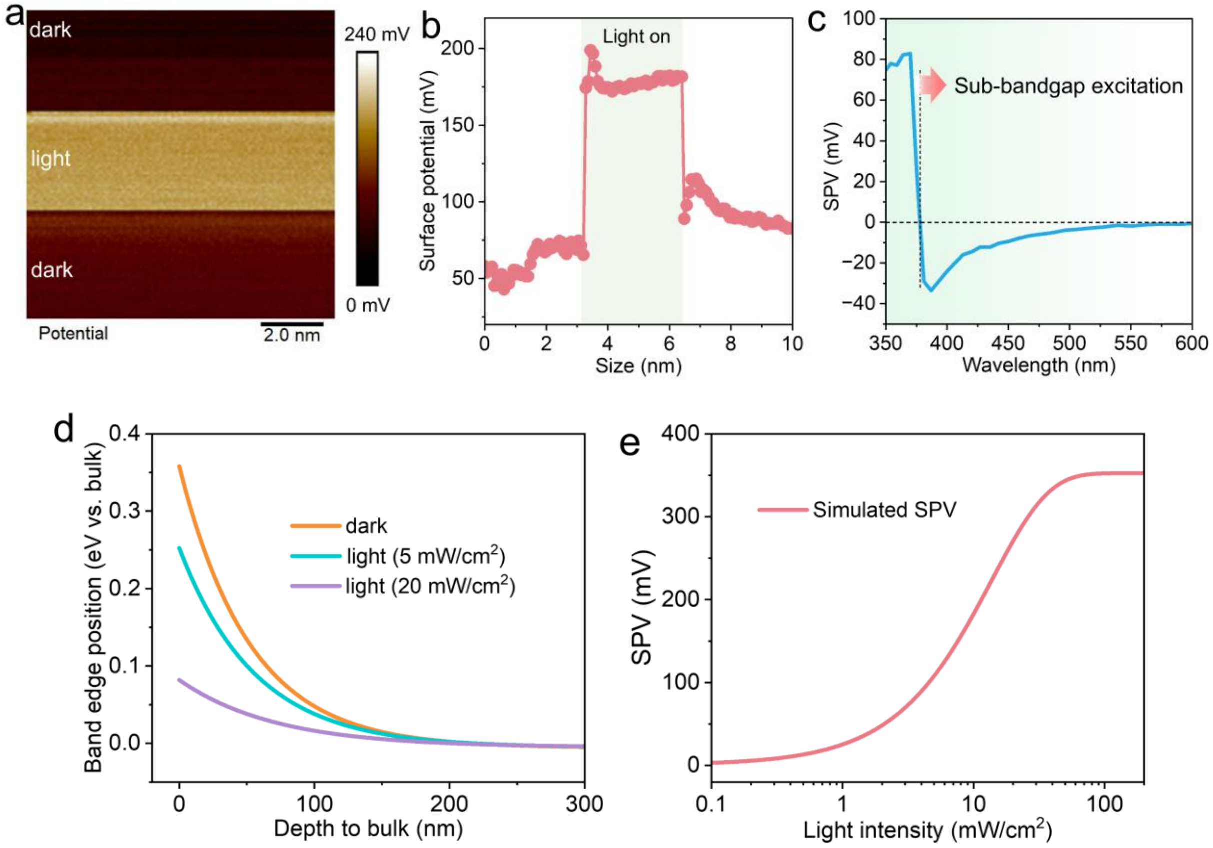

Figure S5. (a) Surface potential measurement on bare GaN surface in the dark and under 380 nm illumination. (b) Surface potential profile of bare GaN in the dark and under 380 nm illumination. (c) Surface photovoltage (SPV) as a function of excitation wavelength for GaN. The positive SPV at bandgap excitation suggests an n-type GaN surface with surface band bending in the space charge region. The negative SPV observed at sub-bandgap excitation is

associated with electron trapping at surface defects located below the Fermi level. (d) Band bending profiles at the GaN surface in the dark and under illumination, illustrating the band-flattening effect induced by photoexcitation. (e) Light-intensity-dependent SPV curve for the GaN surface under bandgap excitation, showing that the SPV increases with illumination intensity and gradually saturates.

We carried out simplified device-level simulations of the band bending and surface photovoltage (SPV) at the GaN surface under illumination. Our approach is based on solving the one-dimensional Poisson-drift-diffusion equations, which describe electrostatics and carrier transport in n-type GaN.

Specifically, the electrostatic potential ($\varphi(x)$) was obtained from Poisson's equation:

$$\frac{d^2\varphi(x)}{dx^2} = -\frac{\rho(x)}{\varepsilon}$$

where $\rho(x) = q(p(x) - n(x) + N_D^+ - N_A^-)$ is the local space charge density, q is the elementary charge, and ε=9 is the dielectric constant of GaN. N_D^+ and N_A^- denote the densities of ionized donors and ionized acceptors, respectively. The electron and hole current densities are described by the drift-diffusion equations:

$$J_n(x) = q\mu_n n(x)E(x) + qD_n\frac{dn(x)}{dx},$$

$$J_p(x) = q\mu_p p(x)E(x) - qD_p\frac{dp(x)}{dx},$$

where $n(x)$ and $p(x)$ are the electron and hole concentrations, μ_n and μ_p are carrier mobilities, D_n and D_p are charge diffusion coefficients, and $E(x) = -d\varphi(x)/dx$ is the electric field distribution. In this coupled framework, Poisson's equation links the local potential to carrier densities, while the drift-diffusion and continuity equations determine how photogenerated carriers redistribute and screen the built-in field. Thus, these equations allow us to calculate

both the equilibrium band bending (dark state) and the modified potential profile under illumination.

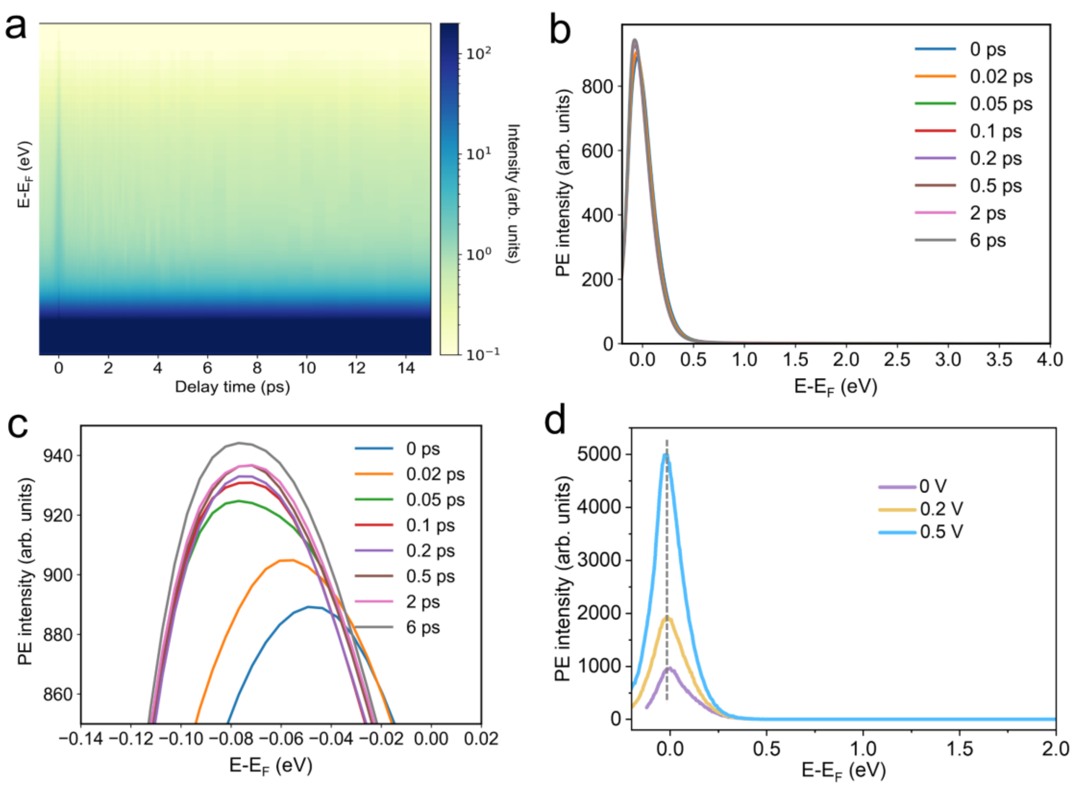

Figure S6. (a) Representative pseudo-color tr-2PPE spectra of GaN as a function of pump-probe delay time, excited and probed by 4.49 eV (276 nm). (b) 2PPE spectra of GaN recorded at different delay times. (c) Enlarged view of photoelectron emission peaks at various delay times. (d) 2PPE spectra of GaN measured at different applied voltages.

The 2PPE spectra recorded at different delay times reveal that the time-independent one-photon photoemission (1PPE) signal exhibits significantly higher intensity than the time-dependent two-photon photoemission (2PPE) process, indicating a high density of occupied states near the Fermi level. As the applied potential increases, the prominent photoemission

peak near the emission threshold maintains a symmetric profile and exhibits no shift in energy position, confirming that this feature predominantly arises from 1PPE processes associated with Fermi-level states. This observation further suggests that the peak position is less influenced by secondary scattering electrons, which typically become prominent at higher applied voltages. Consequently, the observed reduction in the photoemission peak energy with increasing delay time can be directly attributed to the temporal evolution of the surface photovoltage (SPV) of the GaN sample under ultrafast laser excitation.

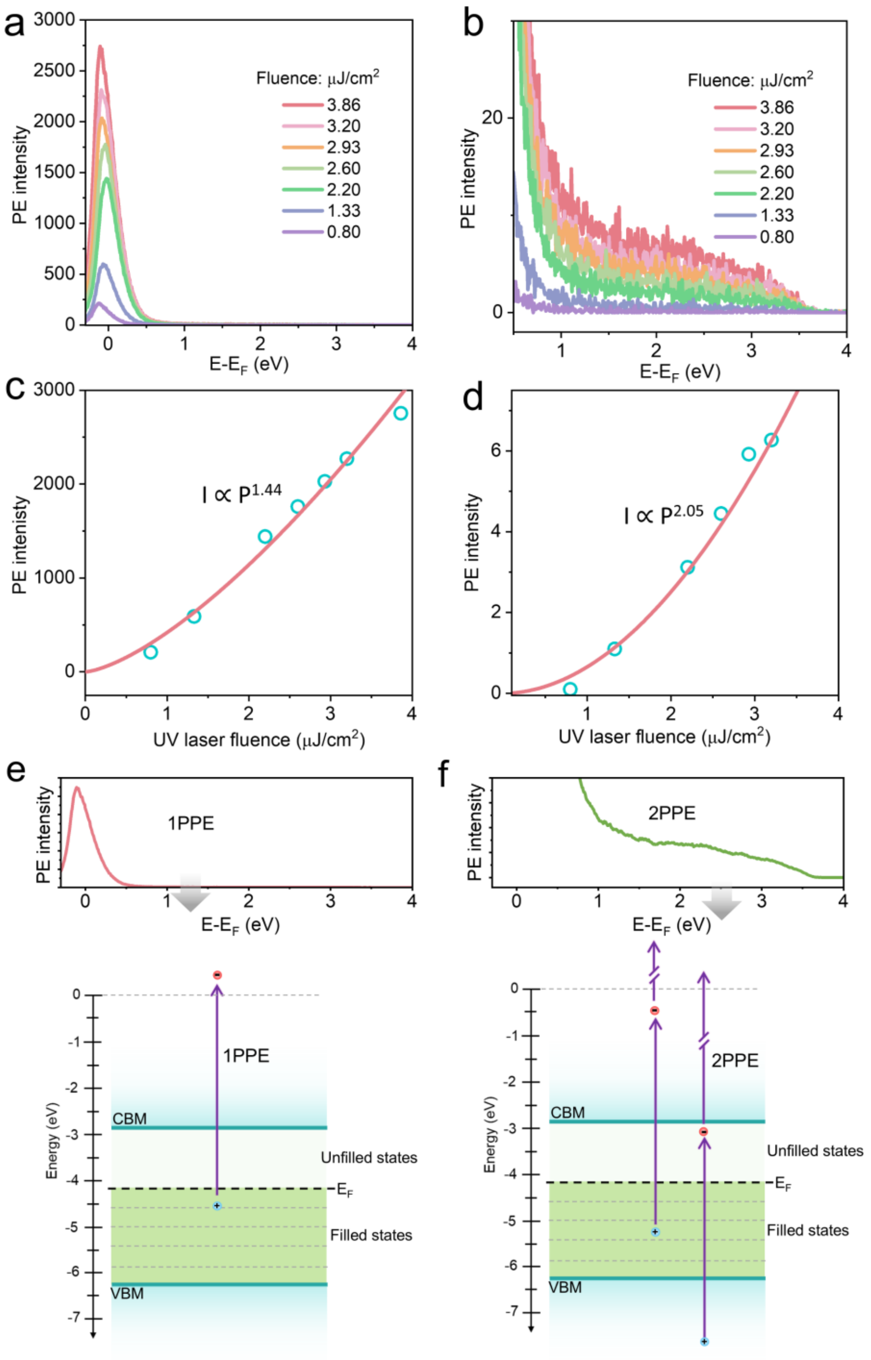

Figure S7. (a) 2PPE spectroscopy of GaN under different UV laser fluences. (b) Enlarged view of the high-energy region of the excited electrons. (c,d) 2PPE intensity (open circles) as a function of UV laser fluence for GaN at $E\text{-}E_F = 0$ eV (c) and $E\text{-}E_F = 1.5$ eV (d). The solid lines represent power-law fits. The fitted exponent of 1.44 for the lower energy electrons indicates the combined contribution from one-photon photoemission (1PPE) originating from the Fermi level and two-photon photoemission (2PPE) processes. In contrast, the signal at higher electron energies is dominated by the 2PPE process from occupied surface states lying below E_F. (e,f)

2PPE spectroscopy and schematic illustration of the corresponding 1PPE and 2PPE processes in GaN.

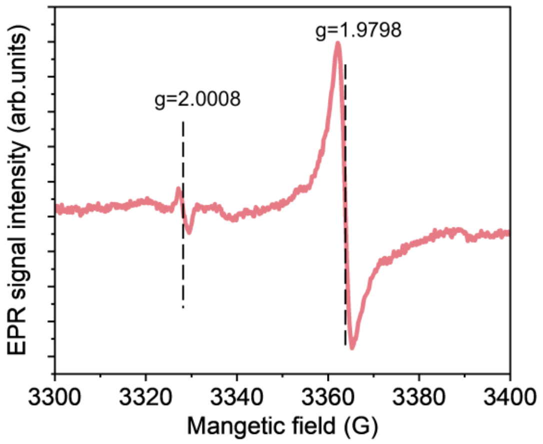

Figure S8. Electron paramagnetic resonance (EPR) spectra of GaN sample. The pronounced signal at a *g* value of 1.9798 is attributed to deep donor states associated with nitrogen vacancies, while a weaker signal at a *g* value of 2.0008 corresponds to shallow acceptor states induced by Mg doping.

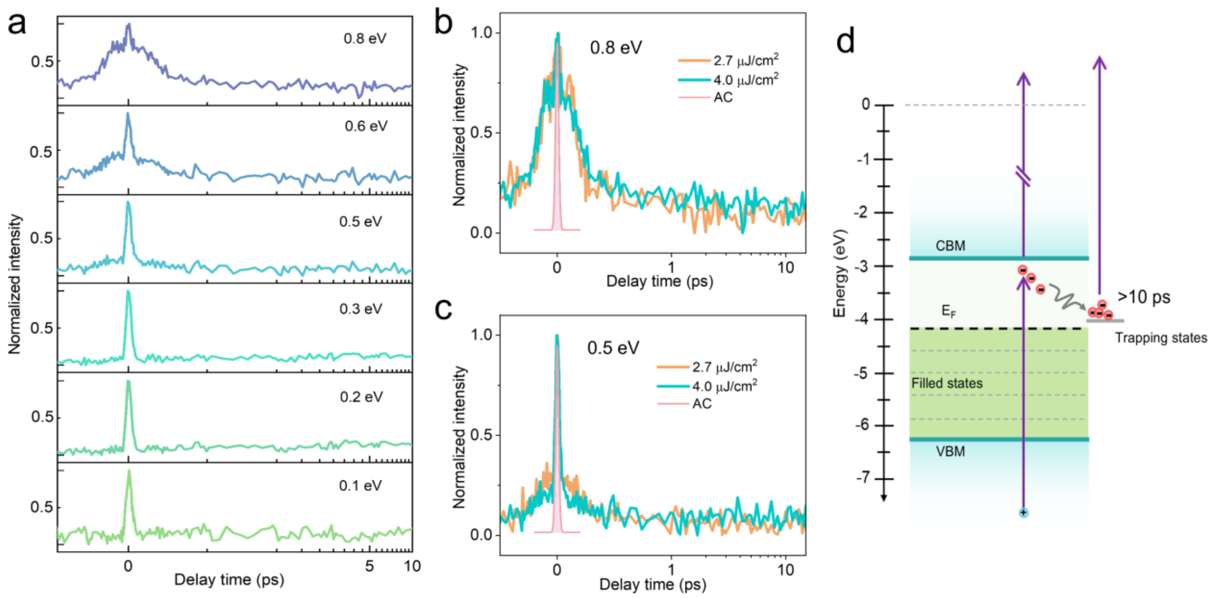

Figure S9. (a) Energy-resolved 2PPE temporal evolution of excited electrons within the defect band below the CBM of GaN. (b) Comparison of 2PPE signal dynamics at an energy of 0.8 eV under different laser fluences. The filled pink line represents the auto-correlation (AC) of pump-probe pulses. (c) Comparison of 2PPE signal dynamics at an energy of 0.5 eV under different laser fluences. (d) Schematic illustration of photoelectron emission from unfilled surface states located below CBM and surface trapping states.

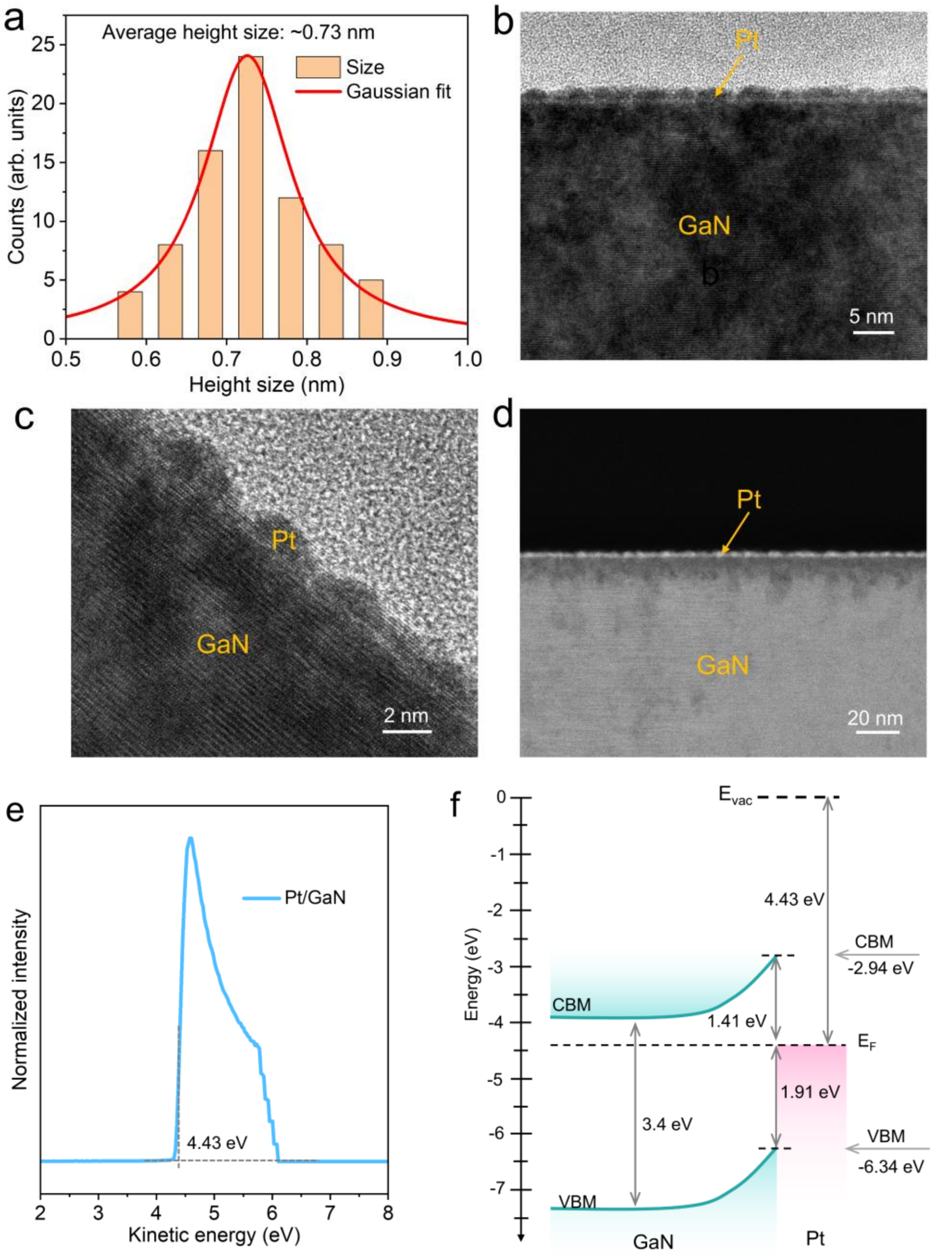

Figure S10. (a) Statistical analysis of height size distributions of Pt nano-islands and the Gaussian fits based on AFM measurements. (b,c) Transmission electron microscopy (TEM) images of the cross-sectional Pt/GaN interface. (d) High-angle annular dark-field scanning transmission electron microscopy (HAADF-STEM) image of Pt/GaN interface. (e) Work function edges in UPS for Pt/GaN sample. (f) Energy band alignment diagram of Pt/GaN sample.

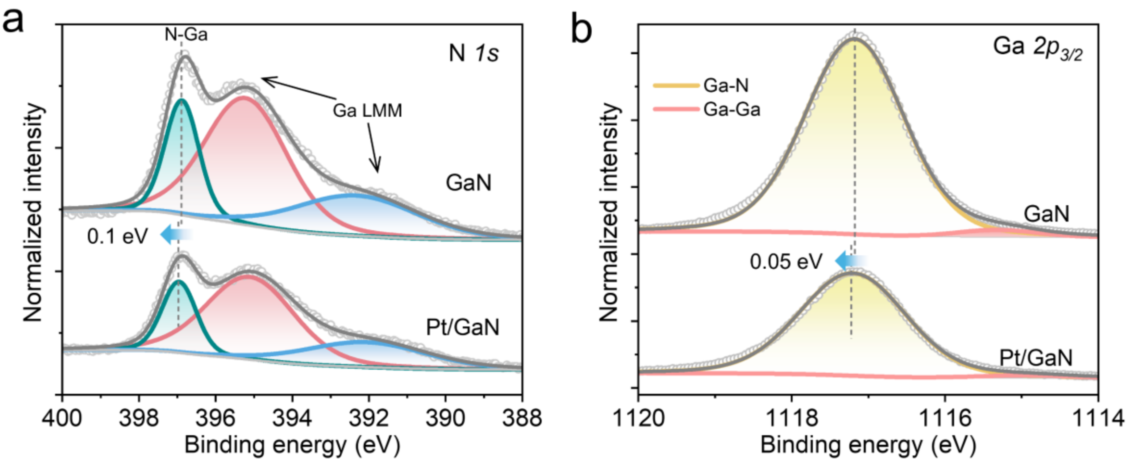

Figure S11. High-resolution XPS spectra of (a) N 1*s* and (b) Ga 2*p* for GaN and Pt/GaN. The dashed grey lines serve as visual guides. The blue arrow highlights the binding energy shift induced by surface Pt modification. The observed increase in binding energy signifies enhanced positive charge accumulation in the GaN substrate after Pt nanoparticle deposition, indicating electron transfer from GaN to Pt. This result suggests the formation of an efficient contact interface between Pt and GaN.

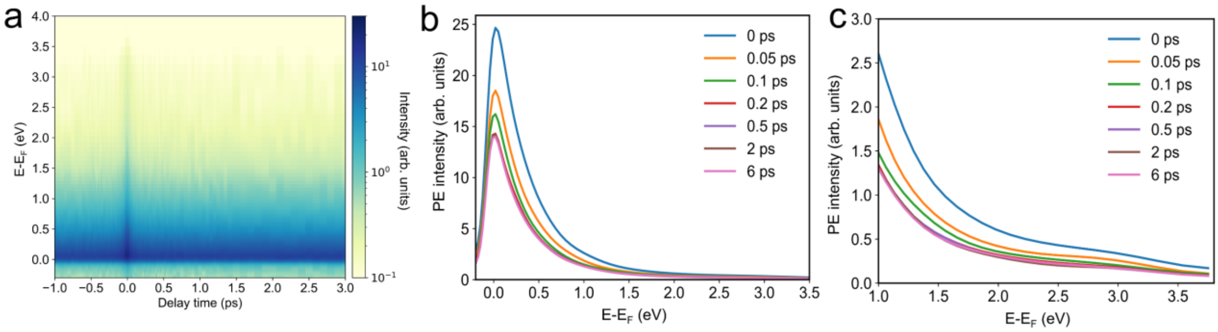

Figure S12. (a) Representative pseudo-color tr-2PPE spectra of Pt/GaN as a function of pump-probe delay time, excited and probed by 4.49 eV (276 nm) photons. (b) 2PPE spectra of Pt/GaN recorded at different delay times. (c) Enlarged view of high-energy electron at various delay times.

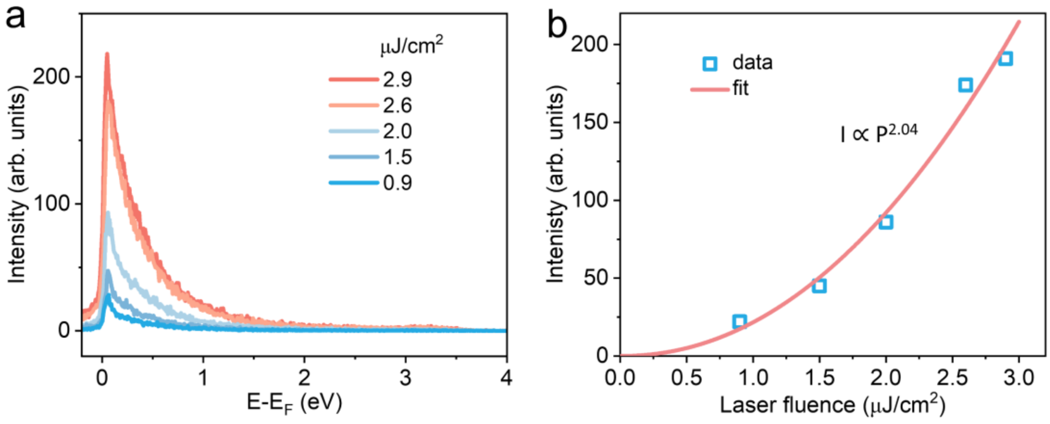

Figure S13. (a) 2PPE spectroscopy of Pt/GaN excited by UV beam at different UV laser fluences. (b) 2PPE intensity (open circles) as a function of UV laser fluence for Pt/GaN at E-E_F = 0.25 eV. The solid line represents power-law fit.

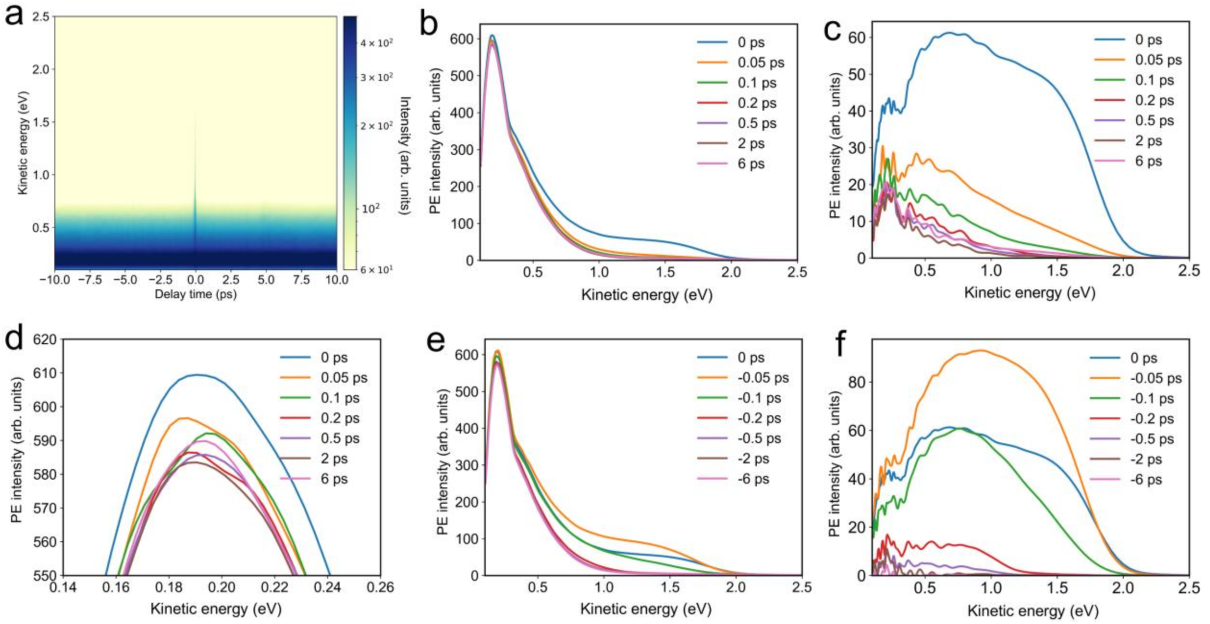

Figure S14. (a) Representative pseudo-color tr-2PPE spectra as a function of pump-probe delay time for GaN using two-colour tr-2PPE. Positive delay time indicates that the Vis pulse reaches the sample surface before the UV pulse, meaning that the sample is excited by visible light and photoemission is induced by UV beam. In contrast, negative delay time represents the opposite case, where the UV pulse arrives prior to the Vis pulse. The energy of Vis pulse is 2.58 eV (480 nm) and the energy of UV pulse is 4.49 eV (276 nm). (b) 2PPE spectra for GaN at different positive delay times. (c) Time-dependent 2PPE spectra for GaN at different positive delay times after subtracting background signal taken at -8 ps. (d) Enlarged view of photoelectron emission peaks at different delay times, illustrating no peak shift with delay time. (e) 2PPE spectra for GaN at different negative delay times. (f) Time-dependent 2PPE spectra for GaN at different negative delay times after subtracting background signal taken at -8 ps.

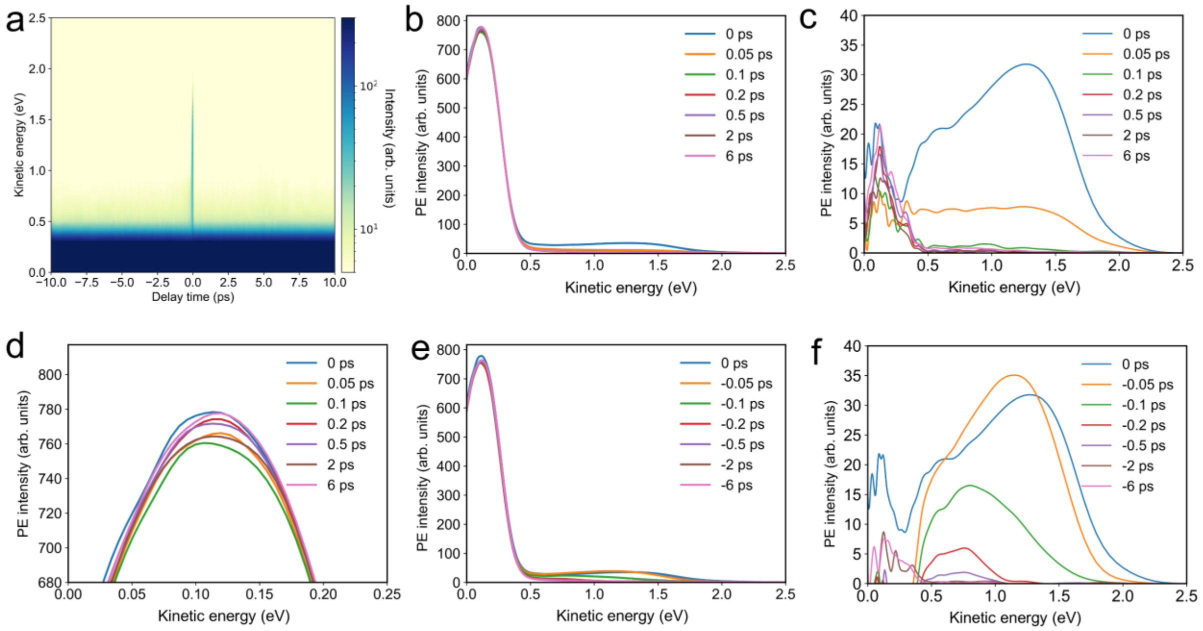

Figure S15. (a) Representative pseudo-color tr-2PPE spectra as a function of pump-probe delay time for Pt/GaN using two-colour tr-2PPE. The energy of Vis pulse is 2.58 eV (480 nm) and the energy of UV pulse is 4.49 eV (276 nm). (b) 2PPE spectra for Pt/GaN at different positive delay times. (c) Time-dependent 2PPE spectra for Pt/GaN at different positive delay times after subtracting background signal taken at -7 ps. (d) Enlarged view of photoelectron emission peaks at different delay times. (e) 2PPE spectra for Pt/GaN at different negative delay times. (f) Time-dependent 2PPE spectra for Pt/GaN at different negative delay times after subtracting background signal taken at -7 ps.

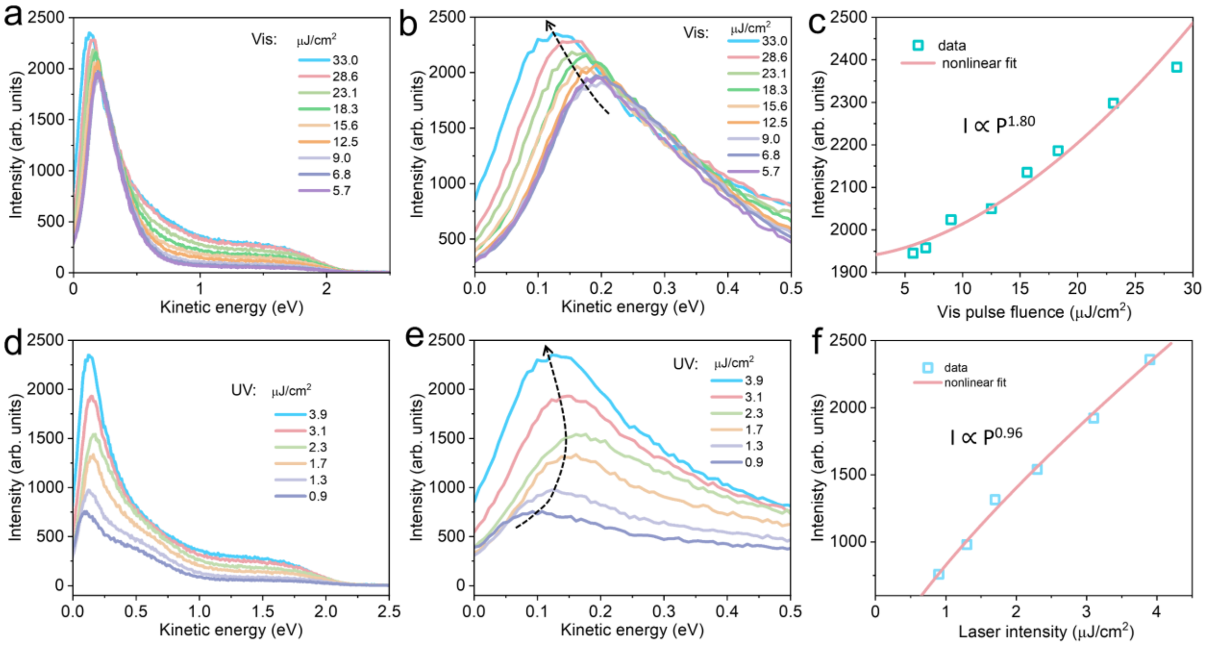

Figure S16. (a) Two-color 2PPE spectra for the GaN sample with varying visible (Vis) pulse fluences while keeping the UV pulse fluence constant at 3.87 μJ/cm^2 (4.49 eV). (b) Enlarged view showing the shift in the photoelectron emission peak with varying the Vis pulse fluences. The dashed arrow illustrates the peak shift. (c) Photoelectron emission intensity (open squares) at kinetic energy of 0.05 eV as a function of Vis laser fluences. The solid line represents the power-law fit. (d) Two-color 2PPE spectra for the GaN sample with varying UV pulse fluences while keeping the Vis pulse fluence constant at 33 μJ/cm^2 (2.58 eV). (e) Enlarged view showing the shift in the photoelectron emission peak with varying the UV pulse fluences. The dashed arrow illustrates the peak shift. (f) Photoelectron emission intensity (open squares) at kinetic energy of 0.05 eV as a function of UV laser fluences. The solid line represents the power-law fit.

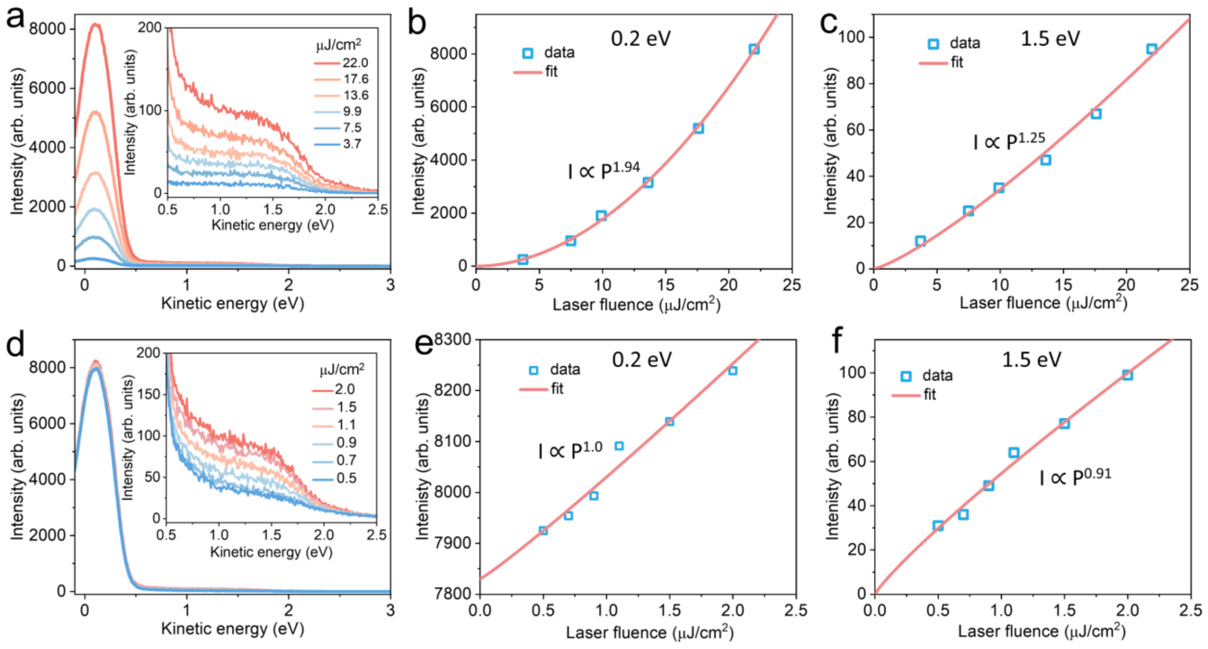

Figure S17. (a) Two-color 2PPE spectra for the Pt/GaN sample with varying Vis pulse fluences while keeping the UV pulse fluence constant at 2.7 μJ/cm^2 (4.49 eV). The inset is a magnified view of the high-energy region. (b,c) Photoelectron emission intensity (open squares) as a function of laser fluences at the kinetic energy of 0.2 eV (b) and 1.5 eV (c). The solid line represents the power-law fit. (d) Two-color 2PPE spectra for the Pt/GaN sample with varying UV pulse fluences while keeping the Vis pulse fluence constant at 22 μJ/cm^2 (2.58 eV). The inset is a magnified view of the high-energy region. (e,f) Photoelectron emission intensity (open squares) as a function of UV pulse fluences at the kinetic energy of 0.2 eV (e) and 1.5 eV (f). The solid line represents the power-law fit.

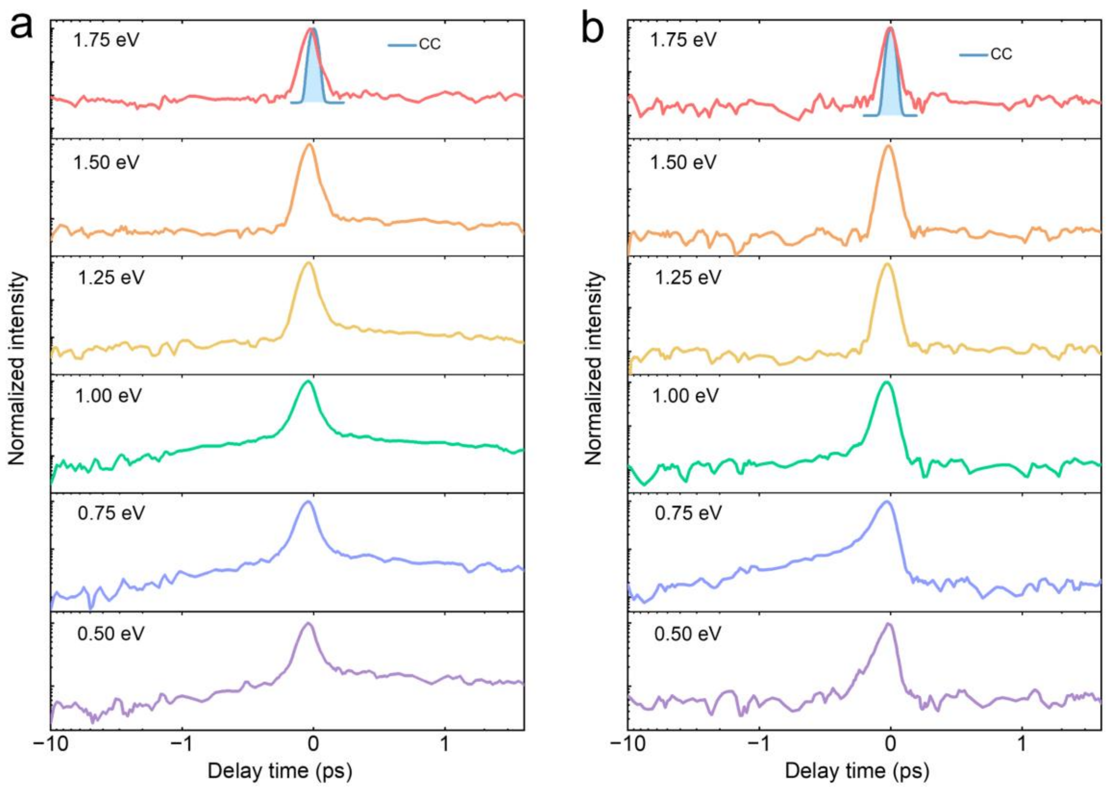

Figure S18. (a,b) Normalized photoelectron intensity as a function of pump-probe delay time at different kinetic energies for the (a) GaN and (b) Pt/GaN samples. The filled blue lines represent the cross-correlation of the pump-probe pulses.

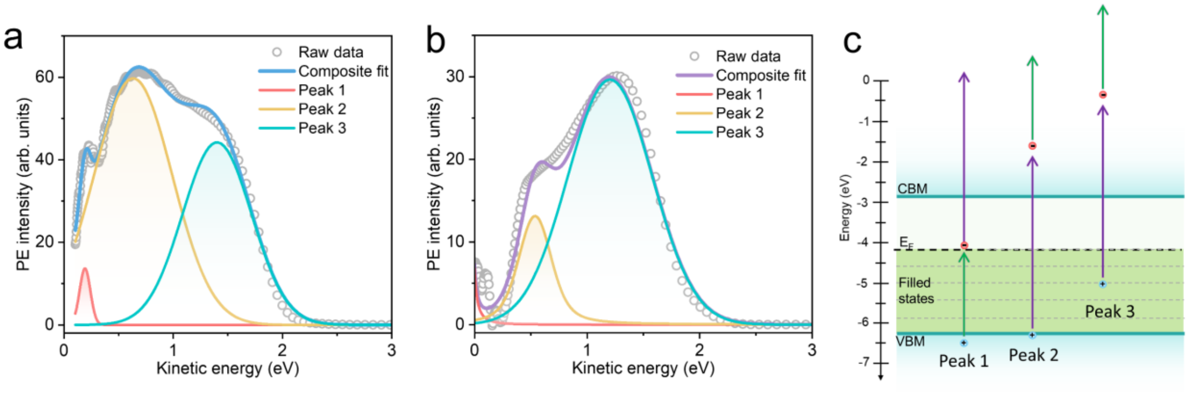

Figure S19. (a,b) 2PPE spectra (open circle) of (a) GaN and (b) Pt/GaN at zero delay time measured at two-colour configuration. The photon energy of visible pulse is 2.58 eV, and the photon energy of UV pulse is 4.49 eV. The lines are Voigt fits to determine the position of each peak. (c) Schematic energy diagram of the three peaks corresponding to two-photon photoemission processes, as determined by the measured kinetic energies and lifetimes.

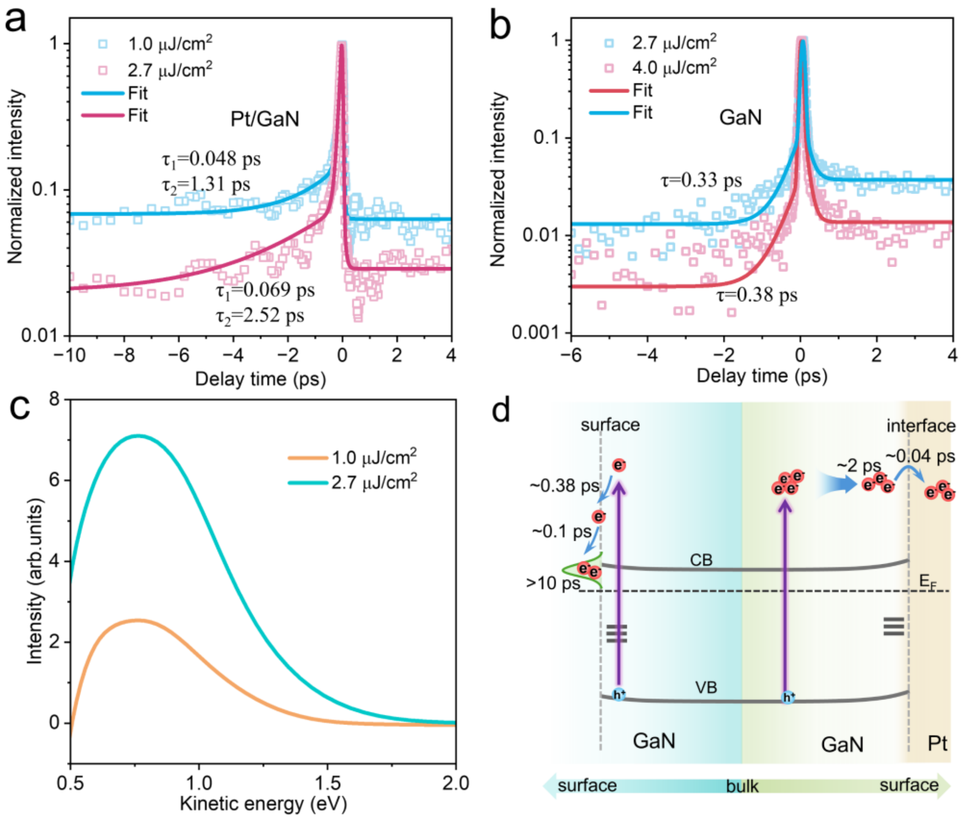

Figure S20. (a,b) Decay dynamics (open squares) of the kinetic energy at 0.75 eV for (a) Pt/GaN and (b) GaN samples with varying UV pulse fluences while maintaining constant Vis fluences (22 μJ/cm^2). The solid lines represent fits to a Gaussian distribution convoluted with an exponential decay function for GaN, and with a double-exponential decay function for Pt/GaN. (c) Time-dependent 2PPE spectroscopy of Pt/GaN at delay time of -0.20±0.05 ps

under different UV fluences. (d) Schematic illustration of photogenerated electron relaxation and carrier dynamics: In GaN, electrons undergo rapid relaxation followed by defect trapping at surface states; while in Pt/GaN, electrons are injected into GaN with energy-dependent behavior, exhibiting suppressed defect trapping, and enhanced bulk-to-surface electron transport due to Pt surface modification.

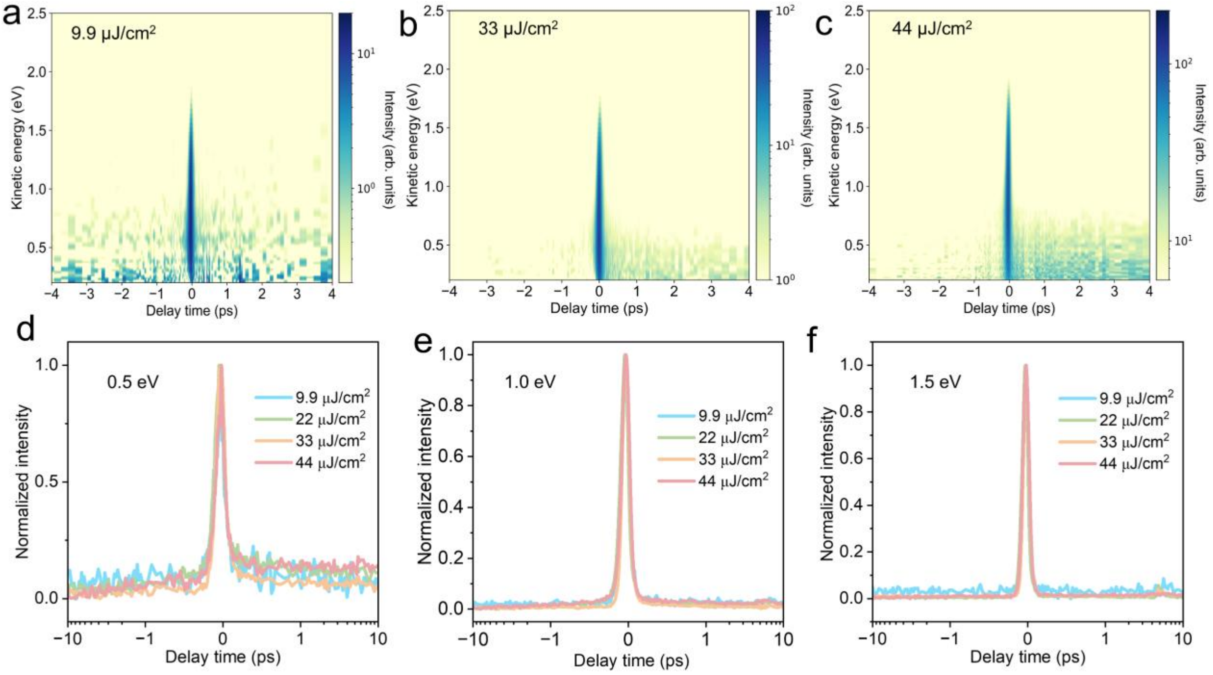

Figure S21. (a-c) Pseudo-colour plot of tr-2PPE spectra of GaN excited at 480 nm with the photon fluences of (a) 9.9 μJ/cm^2, (b) 33 μJ/cm^2, and (c) 44 μJ/cm^2. The wavelength of UV beam is 276 nm. The sample is excited by a visible pulse at positive delay time and by a UV pulse at negative delay time. (d-f) Fluence dependence of the 2PPE signal dynamics for a GaN photoelectrode at kinetic energies of (d) 0.5 eV, (e) 1.0 eV, and (f) 1.5 eV.

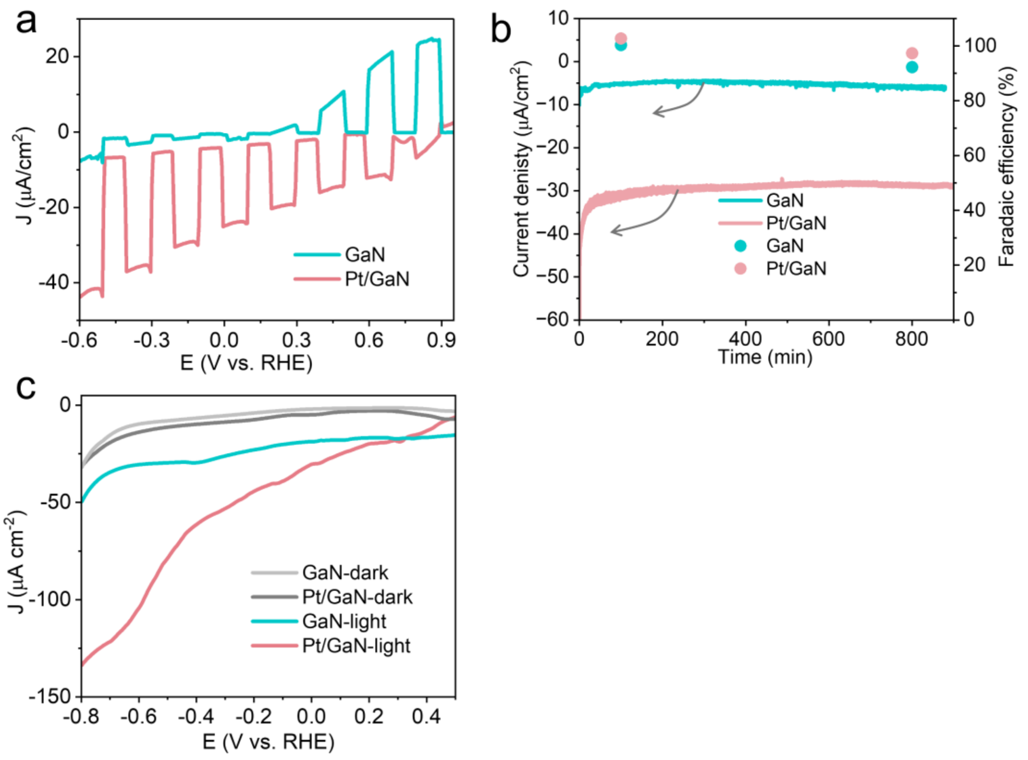

Figure S22. (a) *J–V* plots of bare GaN and Pt/GaN photoelectrodes measured in 0.5 M Na_2SO_4 solution (pH=6.8). (b) Stability test and Faradaic efficiency (solid circles) for GaN and Pt/GaN photocathodes for PEC hydrogen evolution reaction at the applied voltage of -0.4 V vs. RHE. (c) Photocurrent density versus potential curves of GaN and Pt/GaN electrodes in a 0.5 M Na_2SO_4 electrolyte with 5 mM $K_3[Fe(CN)_6]$ (pH= 5.8) in the dark and under illumination.

Table S1. Fitting results for peak energy distribution and the corresponding pump-probe configurations.

Sample	Peak	K_{in} (eV)	Initial state (eV vs E_F)	Pump	Probe
GaN	Peak 1	0.19	2.69	Vis	UV
	Peak 2	0.63	2.25	UV	Vis
	Peak 3	1.40	1.48	UV	Vis
Pt/GaN	Peak 1	-0.05	2.69	Vis	UV
	Peak 2	0.54	2.10	UV	Vis
	Peak 3	1.20	1.44	UV	Vis

References:

1. Florescu, D.I., Asnin, V.M., Pollak, F.H., Molnar, R.J. & Wood, C.E.C. High spatial resolution thermal conductivity and Raman spectroscopy investigation of hydride vapor phase epitaxy grown n-GaN/sapphire (0001): Doping dependence. *J. Appl. Phys.* **88**, 3295-3300 (2000).
2. N'Dohi, A.J.E. *et al.* Micro-Raman Spectroscopy Study of Vertical GaN Schottky Diode. *Crystals* **13**, 713 (2023).
